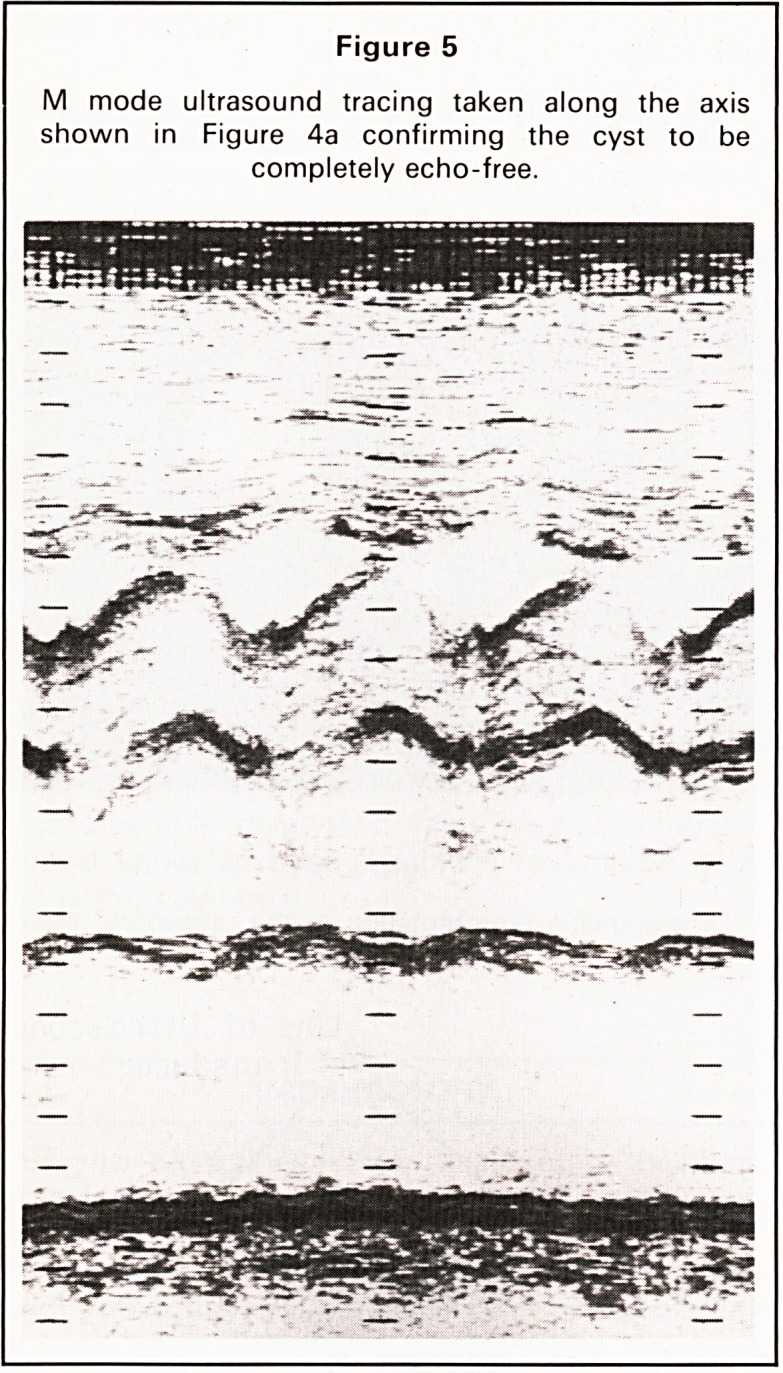# Imaging Techniques in the Diagnosis of a Mediastinal Mass

**Published:** 1983-10

**Authors:** Jonathan M. Bell, J. F. O'Reilly, G. Laszlo, Peter Wilde, Paul Goddard


Case Report

### Imaging Techniques in the Diagnosis of a Mediastinal Mass

**Jonathan M. Bell,* J. F. O'Reilly,† G. Laszlo,† Peter Wilde* and Paul Goddard***
* The Imaging Research Unit, Department of Radiodiagnosis, Bristol Royal Infirmary.
† Respiratory Department, Bristol Royal Infirmary.

### INTRODUCTION

The plain chest radiograph remains the initial and most important investigation in respiratory disease but other radiological imaging techniques are able to provide considerable additional diagnostic information in difficult cases. We present a case in which computed tomography (CT) and ultrasound proved of considerable diagnostic value in explaining an unusual appearance on the chest radiograph of a man with pneumonia.

### CASE HISTORY

A 57-year-old publican was transferred to the chest physicians with a history of pyrexia, productive cough and purulent sputum. He gave a history of chronic productive cough with mucoid sputum since childhood and was a smoker of 40 cigarettes a day for many years. Shortly before referral he had been admitted under a surgical firm with an episode of melaena, investigation of which had shown mild gastritis, oesophagitis and diverticular disease.

Investigation of his respiratory function at the time of referral showed a reduced peak flow rate of 155 litres/minute and an obstructive pattern on spirometry with $FEV_1$ 1.3 litres and FVC 3.15 litres. These results were not significantly changed following administration of a bronchodilator. His sputum grew *Haemophilus influenzae.*

His chest radiograph at that time showed an area of consolidation in the lingula which proved slow to resolve in spite of antibiotic treatment. The lateral radiograph showed a well defined rounded opacity posterior to the heart lying in or adjacent to the mediastinum (Figures 1 and 2). A CT scan of the thorax showed opacification in the lingula due to a localised area of consolidation and a well defined right paramediastinal opacity posterior to the right main bronchus which was thought to represent a neoplasm in the apical segment of the right lower lobe (Figure 3). Bronchoscopy was accordingly performed and was normal.

**Figure 1**

P.A. chest radiograph following resolution of the lingular consolidation. The mass may just be discerned behind the right hilium.

Because of the well defined nature of the lesion and the normal bronchoscopy the possibility of a benign lesion was raised. Two dimensional ultrasound was then performed using the heart as an ultrasonic window. This revealed a well defined transonic structure immediately behind it consistent with a cyst (Figures 4 and 5). The ultrasound also showed myocardial function to be poor. A diagnosis of bronchogenic (bronchial) cyst was therefore made.

**Figure 2**

Lateral chest radiograph taken at the same time as Figure 1 showing the mass as a well defined, rounded opacity behind the heart.

**Figure 3**

CT scan of the thorax showing the mass as a soft tissue opacity lying behind the right main bronchus.

### DISCUSSION

The plain radiograph remains the principal examination in the radiological investigation of chest disease.

**Figure 4a**

Two dimensional ultrasound scan of the mediastinum showing all four cardiac chambers with the cyst appearing as a transonic area with an area of enhancement behind it posterior to the left atrium.

**Figure 4b**

Diagrammatic representation of the ultrasound scan shown in Figure 4a.

However, there are cases in which, by itself, plain radiography is unable to provide a definite diagnosis and other radiographic techniques such as tomography, and more recently, computed tomography are able to provide useful additional diagnostic information.

Radioisotope scanning, either by perfusion or by a combination of ventilation and perfusion, also has a well defined role in certain lung diseases. Pulmonary arteriography may in certain circumstances prove a useful additional tool.

Ultrasound has been used for examining

**Figure 5**

M mode ultrasound tracing taken along the axis shown in Figure 4a confirming the cyst to be completely echo-free.

mediastinal structures, most notably the heart. It has also been used for the assessment of pleural lesions and effusions. Its application in the examination of other mediastinal or paramediastinal lesions by using the heart as an ultrasonic window seems a valuable extension of its potential uses.

Bronchogenic cysts are congenital and benign and those in the lungs are rarely symptomatic unless they become infected.[1] Those which occur in the mediastinum are more likely to be mucus-filled, opaque on chest radiograph and radiologically indistinguishable from other mediastinal tumours. Because of their position and proximity to the large bronchi symptoms may arise as a result of compression or displacement of other mediastinal structures and include exertional dyspnoea, persistent cough or even symptoms of major airway obstruction.[2,3] A cyst may become infected and behave like a chronic lung abscess, producing recurrent fever, productive cough or haemoptysis if rupture into the bronchial tree occurs. Atelectasis due to mediastinal bronchogenic cysts is a rare but well recognised hazard in infants[1,4] but it has also been reported in adults.[5] In the case presented it is possible that the long history of cough is related to compression of an airway by the cyst and the presence of the cyst may also have contributed to the persistence of the pulmonary consolidation.

The differential diagnosis of this mass included bronchial carcinoma and a pericardial cyst. The former was excluded by the good definition and transonic appearance on ultrasound, and the latter by the cyst's position on CT and ultrasound scans.

The management of a patient with a bronchogenic cyst includes elective surgical resection to prevent the development of infection and compression of other mediastinal structures. In the case presented surgical management was relatively contraindicated by his poor myocardial function, and a decision was taken to observe the patient's progress by regular clinical follow up and ultrasound examination to assess the size and position of the cyst. Should the cyst enlarge and cause increasing symptoms by compressing mediastinal structures, percutaneous transthoracic fine needle aspiration would provide a relatively non-traumatic alternative to surgery.

### ACKNOWLEDGEMENTS

The authors are grateful to Miss J. Hugh for secretarial assistance.

### REFERENCES

1. HALLER, J. A., GOLLADAY, E. S., PICKARD, L. R., TEPAS, J. J. III, SHORTER, N. A. and SHERMETA, D. W. (1972) Surgical management of lung bud anomalies. *Annals of Thoracic Surgery* **14**, 434–439.
2. OCHSNER, J. L. and OCHSNER, S. F. (1966) Congenital cysts of the mediastinum, 20 years experience with 42 cases. *Annals of Surgery* **163**, 909–920.
3. DELARUE, N. C., PEARSON, F. G., COOPER, J. D., TODD, T. R. J., ILVES, R. and SANDERS, D. E. (1981) Developmental bronchopulmonary disease in adults – practical clinical consideration. *Canadian Journal of Surgery* **24**, 23–31.
4. STONER, J. and KIRAGUS, C. (1957) Considerations on an unrecognised mediastinal cyst. *Journal of Paediatrics* **51**, 194–196.
5. IKARD, R. W. (1972) Bronchogenic cyst causing repeated left lung atelectasis in an adult. *Annals of Thoracic Surgery* **14**, 434–439.

## Figures and Tables

**Figure 1 f1:**
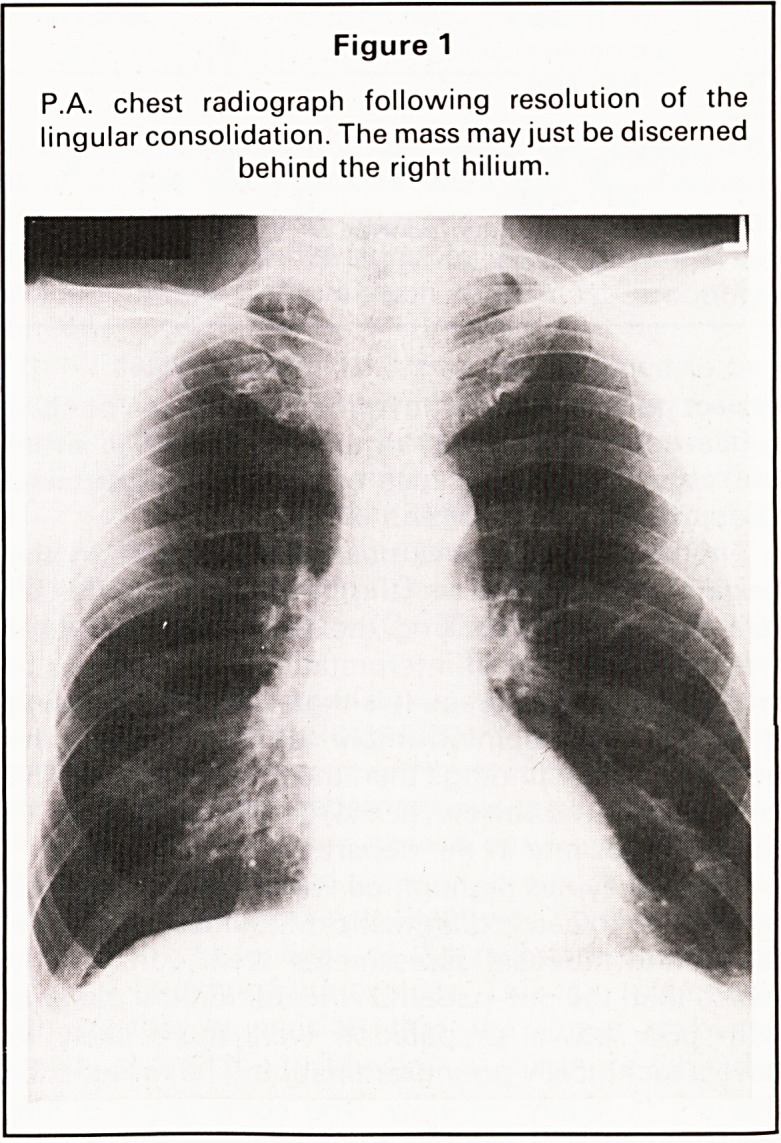


**Figure 2 f2:**
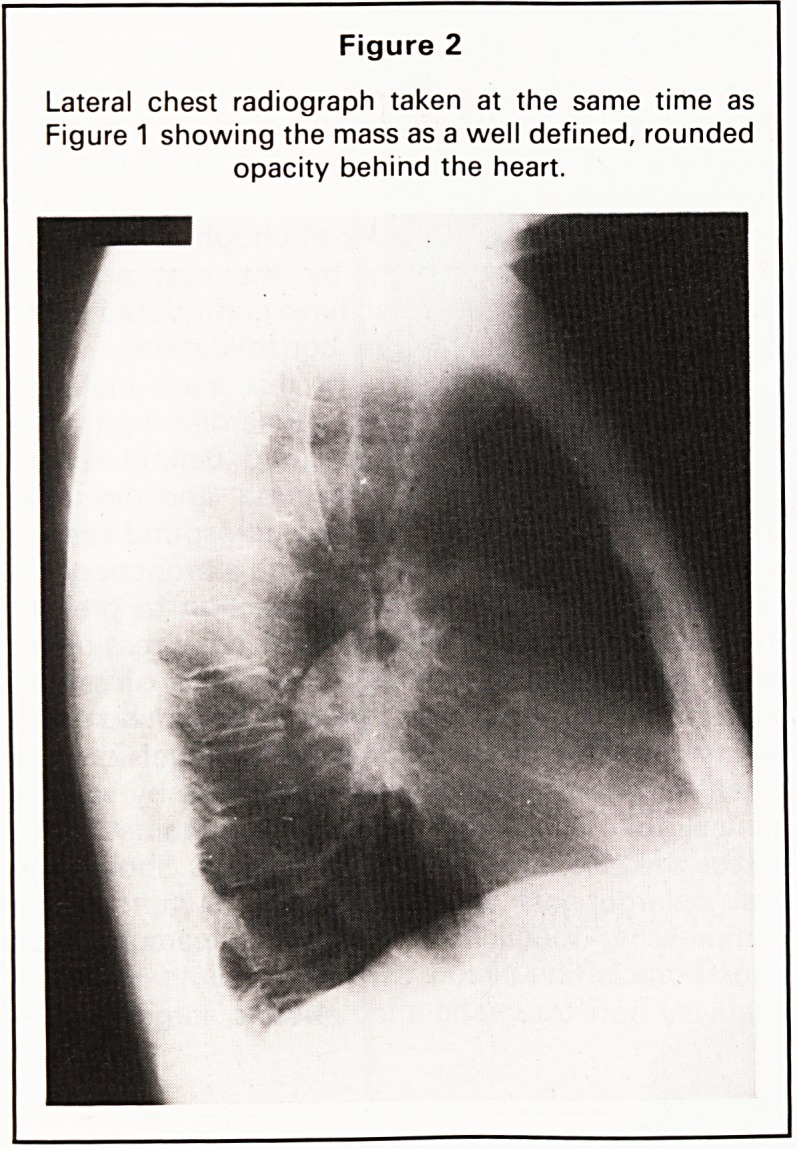


**Figure 3 f3:**
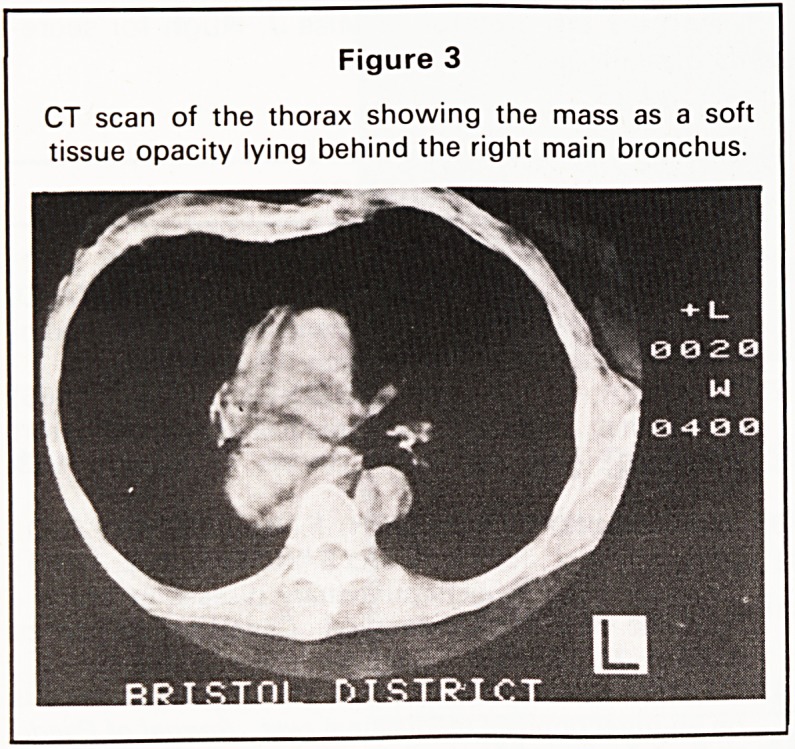


**Figure 4a Figure 4b f4:**
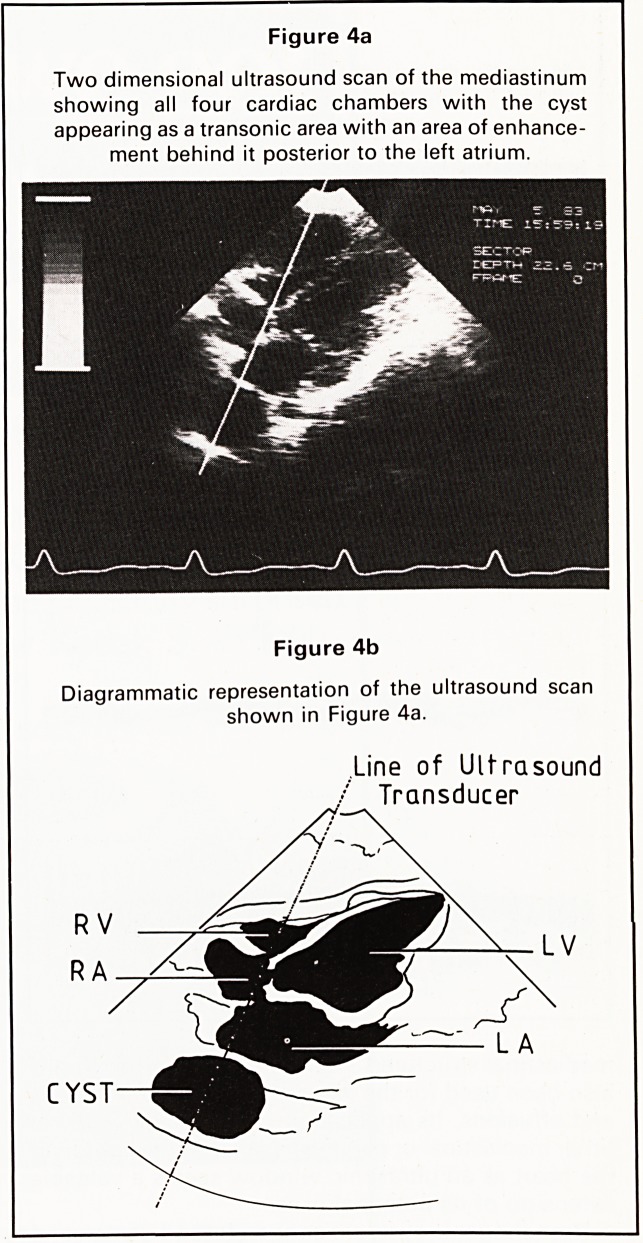


**Figure 5 f5:**